# Victimization and Distress in Indigenous Maya Women: A Qualitative Investigation of Gender-Based Violence and Mental Health Outcomes in Rural Guatemala

**DOI:** 10.1007/s10896-023-00500-2

**Published:** 2023-01-28

**Authors:** Magda C. Rogg, Carla Pezzia

**Affiliations:** Department of Human and Social Sciences, University of Dallas, 1845 E Northgate, Irving, TX 75062 USA

**Keywords:** Gender-based violence, Indigenous mental health, Indigenous women’s health, Emotional distress, Guatemala

## Abstract

**Purpose:**

There is currently scant research exploring Indigenous Guatemalan women’s experiences of gender-based violence and mental health outcomes, but existing research suggests further exploration in this area is necessary. The current study aimed to address this gap by analyzing the experiences with violence and subsequent well-being of Indigenous Maya women in rural Guatemala.

**Methods:**

Data were collected in an ethnographic project on mental health in Panajachel, Guatemala, consisting of a cross-sectional survey on violence exposure and mental health history, followed by semi-structured interviews to elaborate on the experiences. Interviews with seven Kaqchikel Maya women who had been exposed to violence provided the qualitative basis for this study, supplemented by the survey results. Descriptive statistics of survey results and thematic analysis of interviews are presented.

**Results:**

The survey results pointed to a deep relationship between violence victimization and mental health issues. Recurring themes in the interviews included symptoms of post-violence emotional distress including fear, sadness, physical ailments, and suicidal ideation; the role of the women as mothers; lack of legal and mental health support; and the importance of spirituality and religion.

**Conclusions:**

These results highlight the importance of examining emotional distress as separate from diagnostic psychiatric disorders in addressing women’s mental health post-violence. This exploratory study provides examples of violence exposure, mental health, and resource availability among Guatemalan Maya women and suggests possibilities for future investigation.

## Introduction

There is a startling paucity of research concerning the contemporary experiences of and relationship between violence and mental health among Indigenous women in Guatemala. This area lies at the intersection of the study of mental health in Latin America, the mental and emotional well-being of Indigenous women, and Indigenous health in the context of widespread gender-based and family violence, and available research on each of these topics remains scarce (Chmielowska & Fuhr, 2017; Cianconi et al., 2019; Incawayar & Maldonado-Bouchard, 2009; Montenegro & Stephens, 2006; Puac-Polanco et al., 2015). However, since Indigenous women experience high levels of violence globally (Beltrán & Freeman, 2007; Chmielowska & Fuhr, 2017) and violence against women has serious mental health impacts (Devries et al., 2013; Berenzon Gorn et al., 2014; Walker et al., 2019; Grose et al., 2019), there is reason to believe that Indigenous women’s experiences with violence and mental health outcomes are strongly correlated and could pose serious challenges to public health and safety. This applies especially to Indigenous women in rural Guatemala, the focus of the current study, who experience high rates of violence and low access to health resources (Beltrán & Freeman, 2007; Branas et al., 2013; Chomat et al., 2014; Harvey et al., 2010; Menjívar 2008a, b).

### Violence Against Women in Guatemala

Gender and race relations in Guatemala take place within an environment of normalized, “structural” violence, which ran rampant during the 36-year internal conflict and continues to affect the daily lives of Guatemalans (Menjívar 2008a, b). Rates of interpersonal violence have fluctuated in the decades since the conflict, reaching a high of 45.1 homicides per 100,000 people in 2009 and a more recent lower, yet still high, rate of 26.1 homicides per 100,000 people in 2017 (UN, 2019). Once categorized as one of the most violent countries officially at peace in the world (United Nations Development Programme, 2007), the country is currently ranked within the least peaceful quartile of the world’s independent states and territories (Institute for Economics & Peace, 2020, p. 9). Recent qualitative research has provided a platform for Guatemalans to describe their experiences with normalized violence, reporting common themes of physical aggression, violence, and intra-familial conflict (Blucker et al., 2017). While there has been extensive work looking at the impacts of the civil war on mental health, more recent work examining contemporary experiences of violence remains limited.

The violence that pervades Guatemalan society has a strong gender element. Related research notes the role that the cultural orientation of “machismo” (the patriarchal gender norms granting male domination over women) plays in exacerbating intimate partner violence (IPV) among indigenous communities in the highlands of Guatemala (Cepeda et al., 2021; Wands & Mirzoev, 2022). “Marianismo”, or the female counterpart to machismo, places a high cultural value on female sexual purity and further contributes to an environment that values male sexual pleasure over female sexual pleasure and promotes the idea of female passivity and subjugation during sex (Heise et al., 2019). Thus, values such as marianismo and machismo promote a culture of sexual violence and victim blaming in Latin America in general (Castillo et al., 2010; Heise et al., 2019), and notably in Guatemala (de Gamalero et al., 2014). Power differentials between men and women, pressure to stay in a relationship despite abuse, passivity and submissiveness in women, and widespread violence as a method of communication have become common in many Latin American countries and are conceivably the primary motivator of the steadily high levels of patriarchal violence in Latin America (Rondon, 2003).

Guatemala has been called “the most dangerous place for women in all of Latin America” (Suarez & Jordan, 2007, p. 1). Systemic impunity, historical gender inequality, and the normalization of violence as a social device place Guatemalan women in a “vortex of violence” (Carey & Torres, 2010). The most extreme case of violence against women is femicide, used here to mean a killing that appears to be gender motivated (Carey & Torres, 2010). Guatemala has the third highest rate of femicide in the world, and even as the general homicide rate declines, the rates of femicide are declining slower than the rates of murders with male victims (Musalo & Bookey, 2013). Additionally, domestic violence (DV) is “inextricably linked” to femicide in Guatemala (Musalo & Bookey, 2013, p. 273), as shown in the figures that 48% of homicides with female victims occur in their own home, and that 31% of female homicide victims in 2006 had previously filed at least one report of abuse (Borreguero, 2010). Importantly, recent research demonstrates how some policies, like lockdowns during the COVID-19 pandemic, are directly connected to an increase in DV reporting (Iesue et al., 2021).

The prevalence of sexual assault and harassment; femicide; and intrafamilial, psychological, and economic violence against women in Guatemala is emphasized in recent research (The Advocates for Human Rights, 2017; Menjívar, 2008a, b; Musalo et al., 2010; Musalo & Bookey, 2013). Such reports evidence the presence of symbolic violence – a type of structural violence that exemplifies and perpetuates prejudices – linked to the devastating convergence of institutionalized misogyny and generalized violence in Guatemalan society (Menjívar, 2008a, b). This “normalized” (Musalo et al., 2010, p. 166; Carey & Torres, 2010, p. 142) violence has a centuries-long history at multiple levels, including the home, general society, and government; is exacerbated by impunity; and reinforces the subjugation of women (Musalo et al., 2010). Crimes against women, including DV, often go unreported due to lacking trust in the Guatemalan justice system (Beltrán & Freeman, 2007). High impunity rates for violent crimes against women, generally estimated between 97 and 99% in Guatemala (Musalo & Bookey, 2013, p. 271), and the unreliability of legal, investigative, government, and nongovernment systems contribute to an overall lack of support for women (Musalo & Bookey, 2013; Musalo et al., 2010). As such, Guatemalan women must also contend with feminicide, or femicide where the state is implicated due to the lack of guaranteed safeguards protecting women’s rights.

### Violence Against Indigenous Women

Indigenous women face a unique burden of violence (Musalo & Bookey, 2013). An important consideration is that of intersectionality, which emphasizes the limitations of single-axis thinking and promotes considerations of the ways in which multiple social categories and situations interact and overlap (Berger, 2022; Cho et al., 2013; Crenshaw, 1989). Intersectionality makes clear that class, race, gender, and social origin are simultaneous determinants of mental health, safety, success, etc., and is especially crucial in the study of Indigenous women in Guatemala.

Indigenous women face intersectional discrimination, based on gender, race, and social origin. The Maya and other Indigenous populations make up over 40% of the Guatemalan citizenry (CIA, 2018), but they have historically faced discrimination, marginalization, and racial violence, especially during Guatemala’s genocidal internal conflict, during which the vast majority killed were Indigenous noncombatant civilians (Commission for Historical Clarification, 1999) and Indigenous women were killed almost twice as often as non-Indigenous women (Carey & Torres, 2010). Gender violence has mainly been against Indigenous women, but the official statistics have failed to disaggregate femicide data by race and social origin (Abdullah et al., 2012). Ultimately, the Guatemalan government has failed to effectively safeguard Indigenous women’s right to live free from intersectional violence (Abdullah et al., 2012).

### Post-Violence Mental Health Outcomes for Women

Another cause for concern stems from the strong link between gender violence and mental health conditions. Gender-based violence in low income countries was associated with mental health disorders including suicidal ideation (SI) and behavior and symptoms of depression, post-traumatic stress disorder (PTSD), and eating disorders (Devries et al., 2013; Grose et al., 2019). In the United States, female victims of sexual or physical violence are more likely to experience negative health outcomes such as depression and PTSD symptoms, substance use, and self-reported chronic physical and mental illness, and IPV exposure is strongly associated with some psychiatric disorders, especially among women (Afifi et al., 2009; Coker et al., 2002; Weaver et al., 2007). In a study of Mexican women, IPV victimization was associated with worse mental health, less social support, and more traditional notions of gender roles (García Oramas & Matud Aznar, 2015). The high levels of psychiatric disorders and symptoms post-violence among women in Latin America are associated with social factors stemming from restrictive gender norms. Although national gender-disaggregated data are generally lacking, local research indicated that gender-based violence and other gender inequalities were associated with a large portion of depression (Gaviria & Rondon, 2010).

Although the data linking violence victimization to mental health disorders are strong, a consideration of common psychiatric disorders alone may not tell the whole story of women’s experiences after exposure to violence. Another negative ramification of victimization is emotional distress (*malestar emocional*), which refers to the subjective experience of unspecific symptoms or feelings that collectively contribute to a reduction of general wellbeing without meeting the diagnostic criteria for a psychiatric disorder (Berenzon Gorn et al., 2014). The occurrence of emotional distress was associated with daily worries such as DV, as well as traumatic events such as past or present violence, in a study of women in Mexico City (Berenzon Gorn et al., 2014). Multi-country studies found strong associations between IPV and poor health outcomes including emotional distress, difficulty with daily activities, dizziness, memory loss, and suicidal thoughts and attempts (Ellsberg et al., 2008; Golding, 1999; Spencer et al., 2019).

### Indigenous Women’s Mental Health

Generally, global Indigenous populations, especially Indigenous women, experience low standards of health, associated with deficiencies in areas such as socioeconomic status, diet, violence prevention, and access to culturally appropriate health care services (Gracey & King, 2009; Montenegro & Stephens, 2006). Mental health is also affected. Globally, there is still a grave deficiency in research on the mental health experiences of Indigenous women, but available research shows evidence of serious mental health concerns among that population. A review of 13 studies on Indigenous women, IPV, and mental health disorders showed very high rates of IPV and a high occurrence of mental health disorders in Indigenous women across the globe, seemingly exacerbated by poverty, discrimination, and addiction (Chmielowska & Fuhr, 2017). Physical violence was the strongest predictor of psychiatric disorders, and the most common disorders were depression and PTSD (Chmielowska & Fuhr, 2017). In a study of an Indigenous Kuna population in Panama, there were high overall rates of reported SI, and women and low-income individuals were more likely to report depression (Walker et al., 2019).

If reliable statistics on Indigenous mental health in Latin America, especially specific to women, are scarce, the paucity is even more stark when focusing on the unique example of Maya women in Guatemala (Branas et al., 2013). This is concerning because the limited sources available suggest mental health, especially within the context of violence, as an area of need among that population. In a community-based survey of 46 Guatemalans in Santiago Atitlán, 91% reported they were afraid they might be hurt by violence, and 62% screened positive for PTSD (Harvey et al., 2010). The first nationally representative study of the mental health of Guatemalans still living in Guatemala showed that one in five Guatemalans had experienced a violent event in their lives, and women and Indigenous Maya had greater odds of experiencing negative post-violence mental health outcomes (Puac-Polanco et al., 2015).

Although access to professional health services can improve women’s condition and reduce the risk of further victimization, Indigenous women in Guatemala can face many obstacles to receiving care. In developing countries, the financial burden of medical services on poor families greatly affects women (Sen & Östlin, 2008). Oftentimes a woman will rely on her male partner’s income for the necessary resources to seek professional medical aid; if the woman does not have the resources, she often turns to informal sources or more accessible community support networks (Heise et al., 2019). In countries with strong gender norms and inequalities, there can be barriers at each step that prevent women from receiving necessary care, especially for Indigenous women with low resources and suffering from mental health issues.

In Guatemala, specifically, access to health services that could help suffering men and women are severely limited. In 2002, Hannah Roberts reported that 40% of the country had no state mental health services, and the other 60% was served by only 44 state psychiatrists and 61 state psychologists (Roberts, 2002). A national mental health policy was enacted from 2007–2015 that significantly increased the number of psychologists throughout the country, but overall availability of mental health workers remains low at 47.78 workers per 100,000 people (WHO, 2017). Outside of major urban centers, mental health care access is considered transitory and often dependent on the inconsistent labor of student interns (Pezzia, 2015). Only 18% of Guatemalan Maya people with a mental disorder receive any kind of treatment (mental health, other medical, and/or Indigenous healer) throughout their lifetime (Kohn, 2013), and it is likely that Indigenous women have even lower levels of health service utilization.

### Current Study

In this study, the authors were interested in gaining a more intimate understanding of the contemporary conditions surrounding violence victimization among women of Indigenous background in rural Guatemala. High levels of violence and low availability of justice and recovery resources form a toxic combination in the lives of Indigenous Guatemalan women, but the insufficient available data fail to show the scope of the effects of this combination and the underlying trends that future policy should address. The current study aims to explore the ways in which such an environment threatens the health, security, comfort, peace of mind, interpersonal relationships, and agency of Maya women. Through this study, we intend to present the women’s reports of impunity, recurring violence, distress, insufficient health resources, and other struggles as examples of the forms violence against women takes and the types of circumstances that follow violence in Maya communities.

## Methods

### Study Site

Data were collected as part of a mixed methods ethnographic project on mental health conducted in Panajachel, Guatemala, from May 2010-November 2011. While there have been significant events that have happened since the time of data collection (e.g., socioeconomic shifts highlighting gendered dynamics as a result of the COVID-19 pandemic), recent research underscores the extreme lack of available data on intimate partner and other forms of family violence against Maya women (Cepeda et al., 2021; Wands & Mirzoev, 2022). As such, the data presented in this article helps to address a knowledge gap in the existing literature. Moreover, the second author’s continued affiliation with the community and research participants allows for data interpretation to be updated to reflect current events and contexts.

Panajachel is a Western highland town in the department of Sololá, with a population of approximately 16,000 (INE, 2002). The region is considered one of the safest parts of Guatemala, but experiences of violence, particularly gender-based and family violence, are still common. Prior to the initial fieldwork period, a surge in kidnappings spurred the creation of a municipal security commission. One of their primary objectives was to address issues of violence, but most activities centered around coordinating citizen watch groups with little direct intervention to curb violence. Recently, a series of sexual assaults has prompted a local movement that in 2019 organized its first march for International Day for the Elimination of Violence Against Women (November 25). Like many communities throughout Guatemala, Panajachel has a Municipal Office for Women (OMM) that is intended to provide various services like psychological and legal support for women who experience violence. Panajachel also has a public health clinic that on rare occasion hosts psychology graduate students who provide counseling services as part of their required practicum experience.

Historically, Panajachel was a predominately Kaqchikel Maya rural community. A strong tourism industry began to develop in early twentieth century, and since then, Panajachel has developed into a primary tourism destination attracting both national and international visitors. The town is still majority Maya, though not limited to Kaqchikel, and also hosts a considerable expatriate population. A portion of this population and other long-term tourists are heavily involved in the nongovernmental social service sector. Panajachel is considered a regional hub for many local and international nongovernmental organizations (NGOs) that offer financial, education, and nutritional support. One such organization that opened in 2009 provides legal assistance for women experiencing violence. It sometimes offers psychological support on a less consistent basis. More recently, another NGO created a peer support group for women experiencing IPV.

### Study Design

With the help of three local research assistants, we administered a cross-sectional survey from January to November 2011. The research assistant team included two women and one man, all in their 20 s, and two were Indigenous while one of the women was non-Indigenous. We developed a random sampling matrix imposed on a town map that included all households and businesses. This was done to ensure equal representation of genders since most women were likely to be at home while most men were likely to be working during our data collection hours. On sampled streets, we knocked on all house doors and entered all open businesses. When multiple adults were present in a household, we enrolled the person who answered the door (regardless of gender). When multiple adults were present in a business, we enrolled the person who approached the interviewer first (regardless of gender). Participants needed to be fluent in Spanish or Kaqchikel, and all participants preferred to complete the survey in Spanish. Excluding older adults, Panajachelenses are predominantly bilingual or monolingual in Spanish.

The survey included questions regarding experiences of violence and of mental health issues. Experiences of violence were determined by asking if they had been hit, slapped, kicked, or otherwise harmed by someone ever in their lifetime. Participants who responded in the affirmative were then asked who had harmed them. Participants were also asked about their health status, mental health symptoms, and history of mental health treatment. Mental health symptoms corresponded to criteria following DSM-IV classifications of psychiatric conditions. At the end of the survey, participants were given the option to speak more openly about their responses in a semi-structured interview with the lead investigator, regardless of having had a violent experience or not. We administered 414 surveys, of which 64 were subsequently excluded for being under the age of 18 or not living in Panajachel. Of the included surveys, seven Indigenous women who had experienced violence participated in the follow-up interviews.

In the semi-structured interviews, participants met with the lead investigator, a middle-aged Latina from the United States fluent in Spanish. Participants were asked to elaborate on their survey responses and provide additional information about their mental health and any experiences of violence. All interviews were conducted in Spanish and audio-recorded with participant permission. Each recording was transcribed and reviewed for accuracy. Transcriptions were then translated into English and cross-checked by the authors.

Ethnographic data for the purposes of this article draw upon fieldnotes from interviews and from participant observation with the security commission mentioned previously. Interview notes consisted of setting descriptions and behavioral shifts during questioning. The lead investigator attended weekly meetings of the security commission for approximately six months and served as secretary for the last month. Notes were taken during each meeting and included setting and attendance descriptions, summaries of discussions, and any behavioral observations.

*Ethical Considerations.*

The Institutional Review Board at the University of Texas at San Antonio and the University of Dallas reviewed and granted ethical approval for this study. The local public health director provided community-level approval for the project. Each individual interviewed gave verbal informed consent prior to participating in any research activities. Pseudonyms are used throughout the manuscript to protect participant confidentiality.

Given the nature of the questions in the survey and interviews, psychological and emotional safeguards were determined prior to the start of the research. Participants were provided details regarding the nature of the survey questions as part of the informed consent process. They were told they could refuse to answer any questions they did not feel comfortable responding to, and they were reminded of this throughout the survey. Survey and semi-structured interviews were paused by the interviewer if the participants appeared to be getting emotional, and interviews only continued with participant permission. Other safeguards included direct referrals to available psychiatric and psychological services, as well as local non-governmental organizations that provided legal and other services.

### Analysis

We used SPSS, version 25.0, to calculate descriptive statistics of survey responses. We used a thematic analysis approach for qualitative data analysis. Both authors independently reviewed interview transcripts and coded data for emergent themes. We did not have a list of a priori themes, but we focused coding on passages articulating experiences of violence and mental health. Transcript passages were marked up in Word, then categorized in Excel. We then discussed our theme generation and came to consensus on any discrepancies. Table 1 includes all identified themes. Fieldnotes from interviews and participant observation were reviewed to identify any supporting or contradicting evidence of main findings.

**Table 1:** Summary of Participants’ Experiences

Pseudonym	Violence	Distress	Suicidal Ideation	Support	No Support	Themes
Fabiola	SA	Y	Y	Parent	Friends, MH, Pro	Comparison; Small Community
Rosario	Threat; Intra-Family Violence	Y	Y	Religion, Friends	N/A	Potential; Religion; Small Community
Mariela	SA	Y	N	Paren	Law	Motherhood; Small Community
Luz	Threat; IPV	Y	Y	Children	Husband; Law	Potential; Religion; Witchcraft; Motherhood; Small Community
Susana	SA; Threat; Violence from Teacher	Y	N	MH Pro	Parents	Comparison; Potential; Religion
G ema	IPV	Y	N	None	Law	Comparison; Potential; Religion; Witchcraft; Motherhood; Small Community
Felicia	SA	Y	N	None	Parent	Witchcraft; Motherhood; Small Community

*SA* sexual assault, *IPV* intimate partner violence, and *MH Pro* mental health professional. The final column refers to themes identified during the coding process

## Results

### General Characteristics of Violence

To contextualize the prevalence of violence among Indigenous women in Panajachel, we first examined survey results comparing experiences between men and women, then Indigenous and non-Indigenous. Overall, 14.6% (*n* = 343) of survey respondents reported experiencing some form of physical violence. Men and women experienced violence at similar rates. Indigenous men and women experienced violence at rates in proportion to the survey sample demographics. Of the men who reported having been physically harmed (*n* = 25), 60% of them were harmed by someone unknown or a friend, while of the women who reported having been physically harmed (*n* = 23), only 4.3% reported being harmed by someone unknown or a friend. However, women were much more likely to have been physically harmed by an intimate partner or parent (78.3%; *n* = 23) than men (16%; *n* = 25). Of the 23 women who reported experiencing violence, 19 of them were Indigenous, yet there were no notable differences between Indigenous and non-Indigenous women who responded to the survey. Even though 76% of respondents who had experienced violence (*n* = 50) also reported symptoms indicative of mental health disorder (mostly, symptoms related to depression, anxiety, SI, and alcohol use disorder), only 10% had ever sought any kind of mental health treatment in the past.

Mental health and violence were regular topics of discussion at the security commission meetings. The vast majority of discussions focused on general interpersonal violence; however, there were several instances of discussions on DV. These discussions focused primarily on the intersection of alcohol use disorders (AUD) and IPV. Of note, roughly 30–40 people attended these meetings, and there was never more than four women in attendance at a time (including the lead investigator). During a press conference the commission aired on a local television station (after the lead investigator had stopped attending meetings), the commission leaders announced that DV had been eradicated from Panajachel, without any evidence to support their claims.

### Experiences of Violence from Interviewed Indigenous Women

The seven interviews included in this article each described examples of the violence that is commonplace in Guatemala. Four of the participants – Fabiola, Rosario, Mariela, and Susana—reported being sexually assaulted by men. Of these cases, two of the reported aggressors were acquaintances (Fabiola, Mariela), one was a family member (Susana), and one was a romantic partner (Rosario). Susana also described feeling sexual pressure from male acquaintances, making her feel uncomfortable. Felicia reported a sexual assault by a female employer at the age of 11 or 12. Other types of physical violence were also prevalent, and three participants (Susana, Rosario, Luz) discussed experiences with multiple aggressors. Gema and Luz reported being beaten by their husbands, and Rosario reported physical abuse from her father, particularly for having a boyfriend. The latter two women also experienced significant threats of physical violence: Rosario by community members who accused her of going out with a married man, and Luz by her husband’s former romantic interest, who then sent men to viciously beat Luz.

Time from exposure to violence and frequency of violent events varied among the women. Susana and Felicia were children when they were first assaulted, and they were both in early adulthood at the time of the interview. As noted above, Susana continues to experience sexual aggression, as well as physical violence. Fabiola, Mariela, Rosario, Gema, and Luz were all young adults when they first experienced violence. For both Fabiola and Mariela, they experienced violence a couple of years prior to the interview and no longer had contact with their aggressor after the one event. Rosario, Gema, and Luz had experienced multiple violent events, and the most recent event had been within the year prior to the interview.

From the survey data, Fabiola, Mariela, and Gema did not report any constellation of symptoms indicative of a current psychiatric condition. Fabiola did mention in the interview her previous struggles with SI and AUD. Rosario and Luz expressed SI but no other constellation of symptoms. Susana’s endorsement of symptoms indicates likely general anxiety disorder (GAD) and substance use disorder (SUD). Felicia expressed some symptoms of anxiety, but they were targeted toward her poor physical state. Some of her symptoms may also be suggestive of *susto* (a cultural concept of distress in the DSM-V), but the survey questions did not include a complete evaluation for this condition. Of these seven women, it should be noted that three of them responded negatively to experiences of violence in the survey but then revealed during the interview the details of their violent encounters. We expand in our discussion section on why they may not have initially reported these violent events.

### Mental Health Responses to Violence

The most common theme of post-violence mental health response was nervousness or uneasiness, which showed up in all seven interviews. For example, Fabiola had a confidence about her, and she initially did not report her violent experience in the survey. She initially wanted a follow-up interview to discuss her grandmother with dementia. It was only after that when she approached the lead investigator to discuss her sexual assault and opened up about her uneasiness. She had been raped by a friend while intoxicated, and she reported:

*…then I said, “If this happened to me, something worse can happen to me, right? Another worse thing can happen to me.”*

She also felt uneasy about sharing her feelings with friends and family because:

*…I’m afraid of anyone knowing my secrets besides me and the person. So, I haven’t found a person to tell, but what I’ve done is close myself off, close myself off and cry, because I can’t tell anyone, because I feel that I’ll tell them today and tomorrow they’ll tell people, and I feel like they’ll only look at me like, “That one, what a bad life she has, right?” or something like that.*

Other manifestations of this theme included feeling overwhelmed, worried, and upset; being unable to concentrate because of worries; being distracted; worrying about something similar happening to other women; and feeling uncomfortable in large groups.

The next most frequent themes of emotional distress were anguish, sadness, and desperation, each mentioned in a total of six interviews. Anguish is taken to mean emotional “pain,” and the women described feeling hurt, pained, or suffering emotionally. Rosario, Gema, and Felicia expressed emotional pain stemming directly from the effects of the abuse they experienced. Luz, whose husband would beat the children and refuse to contribute to the household, described that it was painful to observe the condition of her children. Susana said she would not tell others of her assault because it was “too painful,” while Fabiola reported:

*I’ve never told anyone [about the assault], and the truth is that it hurts me.*

The theme of sadness manifested itself in sorrow, loneliness, a feeling of worthlessness, and feeling “depressed.” Also relevant to sadness is the theme of emptiness, as Susana described her experiences after she was raped at age six:

*For me, the truth is I’ve always liked to stay strong, but when I’m alone, sometimes, I’m overwhelmed and I feel sad, empty, feeling like crying, but when I see other people’s smiles that I gave them, then those smiles fill me up when I feel alone. But there are moments when you feel alone and get sad, and sometimes I cry to let out some of what I still hold.*

Susana kept a smile on her face throughout the interview, yet she clutched at her chest as she talked about her feelings of emptiness.

Desperation appeared when women felt overwhelmed or without options, or they did not know what to do. Mariela reported being sexually assaulted by an acquaintance in town:

*These situations, I can’t find a way to let go of them or to say them, right? With everything I’m feeling – because I have too many troubles, and on top of that, this other one comes up.*

The theme of fear appeared in five interviews. Mariela, Luz, and Gema were afraid of further violence from their attacker, such that Mariela and Gema talked with the lead investigator about buying pepper spray or making some kind of similar concoction to defend themselves. Luz was not receptive to the idea. Fabiola was afraid that people would judge her for being a victim of sexual violence, and Susana was afraid that another woman would go through what she did:

*And when I saw a girl alone in the street I would say, “The poor thing. I hope nothing happens to her.” I mean, I always had that fear.*

In three cases, the women reported that these feelings culminated in some form of SI. Fabiola wished to die because of the feelings of shame and worthlessness after she was raped:

*Then I really thought of killing myself, of taking my life, because I thought, ‘What is life worth to me like this, an abused, violated person?’ I mean, that hurt so much, and when I hear of an assault it’s like I immediately go back to that moment.*

Luz felt it would be better to be dead because of problems with finances and with her husband’s abusive behavior. Fabiola and Luz wiped away tears as they talked, but Rosario had to pause to recompose herself several times throughout the interview. She came close to cutting herself after her boyfriend pressured her to have sex when she was unwilling:

*So, I felt awful and didn’t feel any support from him… So, at times, those bad thoughts come to you and you think of other things like suicide and resting in peace.*

Four of the women reported physical symptoms coming from their experiences of violence and abuse. The most notable is Felicia, who reports a chronic illness involving seizures which she says began after she was sexually assaulted, yet her doctors treat her seizures in isolation and do not address her sexual assault. Importantly, all interviews had been conducted in an office space rented by the lead investigator, except for Felicia’s. Felicia did not feel comfortable leaving her house for too long because of her physical condition, and so her interview was conducted in her bedroom where she could lie down if necessary. Other women reported lethargy, fatigue, loss of appetite, headaches, insomnia, and abdominal pain which kept Gema bedridden for days. For example, Luz reported:

*And I can’t fall asleep at night. Right now, it’s been three nights without sleeping, without sleeping one bit. See, last month, there were two nights just like that, and now once again, and now it’s been three nights. And I can’t sleep during the day, I can’t fall asleep. I’d like to close my eyes during the day and sleep. And a headache, so much pain right here.*

## Social Problems Related to Violence

The violence also had social repercussions for the women, especially resulting in distancing themselves from others and mistrusting friends and family. Fabiola was afraid of venting to her friends and family for fear that they would tell her secrets to others, and she decided to distance herself from her old drinking buddies because they were unsupportive after she was assaulted while intoxicated. Rosario was distant from her parents because her father was abusive and her mother blamed her for being unemployed, and so she relied on herself and her female friends for emotional support. Additionally, she had to distance herself from her married male friends for fear of what others might think of her. Susana never told her mother about being assaulted at age six because she was afraid of getting scolded, and she left home at age 12 because she never felt emotionally connected to her parents afterwards. Felicia was scolded by her mother after being assaulted by her employer.

There was also a spiritual element in six of the interviews. Luz, Felicia, and Gema referenced witchcraft or some form of evil force enacted on them or their families. Fabiola and Rosario cited their religious beliefs as the reason they avoided suicidal behavior. As Fabiola said:

*… thanks be to God that my family instilled so much in me about the value of life, values, morals, so that made me remember. And I said, “If I go to kill myself, I’ll be like Judas, and only God knows where he is,” and so I said in my soul, “I don’t want to blaspheme. I want to fix my life.” So, I started to think about Peter. Peter repented, and I said, “Oh my God, what am I doing?” when I was going to do it. So in the moment when I was going to do it, I reacted, I started to shake and sweat, and so I threw down what I was going to kill myself with and instead I ran out and I left.*

Motherhood was also a prominent theme in the interviews. Mariela, Luz, Gema, and Felicia were mothers and each one mentioned being worried for her children. Mariela was worried for her daughter’s health and was stressed because the fear and trauma she was experiencing after the assault was keeping her from spending her time caring for her children:

*I have problems, and not just because of that problem [sexual assault], but I have family problems. I have two children and I have a little girl - because she’s a little ill. And on top of that, this problem comes up that doesn’t let me concentrate at all.*

Gema was abused by her husband when she left the home to work to provide for her children, and she was afraid that her son was beginning to emulate her husband’s habits of drinking, drugs, and abuse. Gema had come across as stoic as she recounted her husband’s abuse toward herself, but she became animated and raised her voice as she spoke of her concerns for her son. Luz was afraid because threats were being made to her children, she lacked the resources to reliably provide for her children, and her husband would beat the children. Her concern for the wellbeing of her children was the main factor that kept her from suicidal behavior, and she described relief from her worries while spending time with her children, watching them play. Felicia was worried for the health of her son, who was struggling in school, and she lamented that her chronic illness frightened her children and kept her from being able to care for them. Her son had entered the room around this point of the interview, and he quietly nodded in agreement.

## Resource Seeking Post-Violent Experience

Many of the women sought resources for support after experiencing violence, often with limited success. Mariela received no response from the police after reporting her sexual assault. The authorities refused to do anything to help Gema free herself from her abusive husband, saying that he must not be abusing her if they continue to live together. A similar sentiment about IPV in general had been expressed by one of the members of the security commission. After Luz’s husband beat their son, she accused him in front of a judge, who ordered that the son be taken to the doctor but did not penalize the father at all. She never reported the woman who sent men to beat her because of threats to her children:

*I didn’t go to make accusations in front of a judge that she threatens me and does things to me, because she threatened me that if I speak up, she’ll kill my children.*

Fabiola and Felicia considered going to a psychologist but found it too expensive. Susana met with a psychologist regularly and those meetings helped her process her emotions:

*The psychologist helps me; now I’m more at peace, because that isn’t going to be like a burden to me that keeps me from moving forward with my life…. Once a week, I was talking with her, because believe me, I hadn’t spoken with anyone… and now it’s different. I spoke to her about that and all the things that have happened to me, and now I’m getting better. I was able to get over those fears, and now I feel a little more whole.*

During the fieldwork period, the security commission had coordinated with two psychology graduate students to provide counseling sessions to groups of children. These students worked with the lead investigator, as well, to provide counseling sessions to interested study participants. Of the women mentioned in this article, Fabiola went to multiple sessions and found them to be beneficial. She lamented when they were no longer able to come to town, but she stated she felt like she had been given good advice she could continue to incorporate into her daily care.

Two of the women who reported evil forces at work in their lives, Luz and Gema, turned to a priest or religious person to help provide spiritual guidance.

Other women chose to turn to themselves or loved ones for support. Fabiola credits her mother for helping her when she was feeling worthless and suicidal, and after the assault she has devoted herself to reading educational books and studying a health profession, but she is otherwise isolated and deals with her emotions by crying alone:

*I haven’t found a person to tell, but what I’ve done is close myself off, close myself off and cry, because I can’t tell anyone, because I feel that I’ll tell them today and tomorrow they’ll tell people… because I remember the moments when I shut myself in and I cry and cry and cry like a child.*

Rosario has never sought therapy, but rather relies on lifting herself out of episodes of distress and talking through her emotions with her female friends. Mariela never told anyone about the assault but her mother.

The security commission mentioned the need for regular mental health care on various occasions. However, it was typically in reference to children, such as when they coordinated the graduate students group counseling sessions. Other times, it was in reference to men suffering from AUD or SUD. Regular mental health services were never discussed in reference to women.

## Discussion

The interviews provide insight into unique post-violence experiences of emotional distress and external factors that influenced the quality of the women’s lives. Firstly, the interviews provided examples of the types of emotional distress a woman can experience after a violent event. Secondly, the interviews revealed three main factors besides the violence that affected the women’s lives in the post-violence period and their ability to cope with the effects of the violence: their role as mothers; impunity and lack of support in the realm of law enforcement; and spirituality and religion.

### Emotional Distress

All seven participants described ongoing symptoms of emotional distress since experiencing a violent event. Yet only three of them had clearly defined associated psychiatric conditions: one with GAD and SUD, two with SI. While it is possible one participant may be experiencing *susto,* her association of epilepsy to her violent event is not a symptom typically associated with the cultural concept of distress (CCD; Weller et al., 2008). The symptoms that all the respondents described corresponded to symptoms of distress mentioned in the study from Berenzon Gorn et al. (2014), including nervousness, anguish, sadness, fear, emptiness, headaches, insomnia, fatigue, and irritability. Studies from multiple countries showed that women were more likely to suffer from these conditions than men and that the conditions were associated with lower quality of life, overall health conditions, and functioning abilities (Rivas et al., 2011; da Silva Lima & de Almeida Fleck, 2007; Backenstrass et al., 2006). Experiences of gender-based violence can become embodied in various physical symptoms or subclinical malaise, and the dynamics of gender and violence in Guatemala provide context for instances of emotional distress, which, examined in isolation, might seem idiosyncratic (Menjívar, 2008a). These findings might help explain the negative physical health impacts – such as nausea (Mariela), muscle or head pain (Gema, Felicia), insomnia (Luz, Gema, Felicia), and lethargy or fatigue (Luz, Susana) – related to the women’s general experiences of emotional distress.

To look at women’s post-violence mental and emotional state through a strictly diagnostic lens, or to pathologize distress by characterizing disorder as the only possible explanation, can oversimplify the causes of distress and fail to address external events or societal trends that contribute to distress (Tseris, 2016), like the examples provided in the section on the social problems related to violence. Oversimplification can discourage patients from telling health care professionals about symptoms of distress that stem from traumatic experiences. Female victims of IPV who seek health services tend to do so to address physical troubles, and it is less likely that they seek professional support for mental health issues such as emotional distress (Tiburcio Sainz et al., 2010). Although these nonspecific symptoms have real effects, patients often do not discuss them during health consultations, and health care professions often ignore, or minimize the importance of, these reports (Berenzon Gorn et al., 2014). The latter is evident in the case of Felicia. She described her belief that stress, frustration, and sadness has worsened or induced convulsive episodes, but the doctors she consulted had neither recommended seeing a mental health professional nor explored the possibility that psychological and emotional stresses could have brought on or aggravated these episodes.

Examination of emotional distress is further limited from a biomedical diagnostic framework that does not account for cultural differences in the expression of and understanding of suffering (Jenkins, 1994). The expression of suffering and emotion varies cross-culturally, and biomedical diagnostic categories such as the DSM which were validated initially for a Euro-American population can lead to the “category fallacy” when applied inflexibly across cultures (Kleinman, 1987). While the DSM-V addresses some of these issues with its inclusion of CCDs, it is still important to examine the lived experience of suffering and its physical and social manifestations through a culturally sensitive and ethnographic lens (Kleinman & Kleinman, 1991). This is apparent in the expression of subclinical distress among our interlocutors, whose suffering was often insufficiently acknowledged and understood by mental health professionals and community members.

### Motherhood and Womanhood

The effects of a culture of marianismo are manifested in the women’s relationship with and understanding of their own womanhood and motherhood in the post-violence period. For Rosario, the pressure for sexual purity came in the form of abuse from her father, who beat her for having a boyfriend, and threats of physical violence from community members who accused her of going out with a married man. In the first instance, although she asserted that her father was too harsh, she believed he was right to get angry at her for having a relationship. In the second, Rosario felt shame and disapproval even from her parents and had to distance herself from her married friends. The feeling of having a blemished womanhood after sexual assault was expressed most explicitly by Felicia, who reached an extremely low point of self-esteem after she realized she had been raped by her friend and considered herself “an abused, violated person.” The concept of moral capital within the marianismo framework contributes to the need for many women to be seen in a positive light by those around them, thus making it difficult for the women to express their pain or distress. Marianismo can exert pressure on women to silently bear any pain or suffering, and any deviance from this pattern can damage a woman’s reputation as the virtuous silent sufferer (Menjivar, 2008a, 18). Furthermore, a woman may feel shame associated with being a survivor of gender-based violence which keeps her from reporting the incident for fear of losing moral capital in the community. On the other hand, a desire for moral capital may result in accepting violence as a form of punishment for deviating from the mores of marianismo, as in the case of Rosario and the physical abuse from her father.

The roles associated with motherhood are intimately linked with emotional distress for the four participants with children and are related to gender norms emphasizing female silence and domesticity. Mariela, Luz, Gema, and Felicia all described and demonstrated distress stemming from struggles their children were facing, and they worried about providing for their children. The cult of domesticity causes mothers to be disproportionately burdened with unpaid domestic work, and the higher emotional strain and time constraints associated with this burden pose challenges to women’s mental health (Gaviria & Rondon, 2010). For example, low-income mothers in Nicaragua experienced maternal mental distress as a result of their responsibility to deal with food insecurity in their households, and their peace of mind was largely dependent on their children’s food security (Piperata et al., 2020). These challenges evidently have strong reflections in a post-violence context as well. Interviews with female domestic abuse survivors suggested that those who had children suffered from a slower recovery from the effects of the abuse, as well as lower levels of subsequent mental and physical health, both real and perceived (Carpiano, 2002), such as in the case of Mariela’s description of caring for her ill daughter after experiencing sexual assault.

A related area that appeared in the interviews was the feeling of shame and silence causing Rosario, Susana, and Felicia to feel uncomfortable sharing their experiences with violence and/or emotional distress with their mothers. This could be due to a culture of marianismo, and it aligns with the findings of a study of Latina mother-daughter dyads in the United States, which showed that gendered roles of silence and suppression could culminate in suicidality (Szlyk et al., ﻿^2019^).

### Access to Resources

Women described their (often failed) attempts at accessing professional legal, law enforcement, or mental health services in the aftermath of violence, with Fabiola and Felicia unable to see mental health professionals, and Mariela, Luz, and Gema failing to secure judicial and police assistance. This mirrors impunity and the lack of integral assistance – “medical, legal, social, psychological, job training, and language assistance” provided to victims – in Guatemala, which persist despite treaties, pledges for action, and domestic laws, including the 2008 *Ley contra el femicidio y otras formas de violencia contra la mujer*, aimed at improving the situation of women (Ruiz, 2018, p. 111; Musalo et al., 2010; Musalo & Bookey, 2013).

These factors also affect the ability of women to obtain preventative measures against further violence. It is common for judges to insist that women should reconcile with their abusive husbands instead of granting a protective order, as reported by Gema, who was denied protection from her abusive husband. Integral assistance is often inaccessible to a large portion of victims, and Indigenous women experience even more extensive obstacles to justice, including distance to courts, language barriers, and discrimination (Musalo & Bookey, 2013; Ruiz, 2018).

### Religion and Spirituality

Lacking reliable professional resources, Fabiola, Rosario, Luz, and Gema described the importance of spirituality and religion in their times of need. Studies show nuanced links between violence, spirituality, religious coping, and mental health outcomes. Although some studies suggest that religiosity can have negative or mixed impacts on mental health (Ake and Horne, 2003; Kaufman et al., 2020; Knickmeyer et al., 2004), some studies imply that positive spiritual coping and religiosity mitigate mental health symptomology (Ano & Vasconcelles, 2005; Jocson & Ceballo, 2020; Mitchell et al., 2006) and that religiosity is associated with lower rates, not of SI, but of suicidal behavior (Jacob et al., 2019; Burshtein et al., 2016; Lawrence et al., 2016). The latter tracks with the experiences of Fabiola and Rosario, who experienced SI but whose religious beliefs helped them avoid death by suicide.

## Limitations

Our findings are subject to three primary limitations regarding measuring violence. The first of these is the timing of the data collection. Because the data were collected in 2011 and given the more recent increase in sexual assaults in the region and noted increases throughout the nation due to the COVID-19 lockdown (Iesue et al., 2021), it is likely that the prevalence of violence toward Indigenous women is greater than what our survey results indicate. Secondly, even though our survey sample demographics were similar to those found in Panajachel, our rates of violence are likely not representative of the nation as a whole since the Department of Sololá is known to be one of the safest regions in the country. Lastly, we believe our numbers reflect under-reporting since we identified at least three women who had experienced violence but did not feel comfortable stating as such during the survey portion. Since two of these women had been sexually assaulted, we believe this demonstrates the regular under-reporting of sexual violence by women. A more systematic sampling process for the qualitative interviews would have allowed for more robust data triangulation to be able to better address data discrepancies.

Additionally, we recognize that some psychiatric disorders are not experienced as a constant state, but rather as dynamic conditions that may be influenced by various external stressors. Our cross-sectional design likely failed to capture temporally related shifts in mental health statuses, such as Fabiola’s SI immediately after her rape. Even more recent SI from any of the participants may have been missed depending on when they were interviewed. For example, Guatemala’s seasonal patterns that includes a heavy rainy season may have an effect on the expression of depression and SI that might not otherwise be present during other times of the year. Similarly, weather patterns have been noted to affect rates of DV, and there is no research that systematically examines these intersections in Guatemala (Iesue et al., 2021).

Last, the influence of the expatriate population and NGO sector in Panajachel is difficult to disentangle from the general experience of Indigenous women in the region. Foreign and Guatemalan born non-Indigenous migrants have been a driving force for initiatives addressing local violence. Furthermore, many of the NGOs in town limit the services they provide to Panajachelenses, but nevertheless there is greater accessibility to resources than in some more remote communities. However, it should be noted that none of the women saw the OMM as a resource, and only one of them (Luz) had sought services at the NGO that helped women experiencing violence. More research is necessary to better understand these dynamics.

## Conclusion

There is currently a dearth in research focused specifically on Maya women’s experiences of violence and the impact on mental and emotional well-being in contemporary Guatemala. Despite this gap, it has become evident that these issues pose a serious threat to a significant portion of the Guatemalan populace, and thus our study aims to help highlight various nuances of the characteristics surrounding violence victimization of a small sample of rural Maya women. We found that these women were subjected to environments replete with (often gender-based) violence and privation; that their experiences with violence deeply affected their mental and emotional well-being, threatened their sense of security, and impacted their interpersonal relationships; that they had limited success accessing the benefits of formal support structures, such as law enforcement and health care, which could have limited the negative effects of violence victimization; and that, in such conditions, informal support structures such as spirituality or social networks were widely utilized. Thus, besides addressing the dearth of research at the intersection of Indigeneity, womanhood, violence, and mental health in Latin America, our research also adds unique findings to the existing relevant literature, particularly by describing the patterns of Indigenous women’s relationships with their own womanhood, motherhood, and spirituality in a post-violence context.

These women’s stories have yet to be fully explored despite historical and contemporary patterns of victimization, privation, and inequality. Although this study details certain areas of need, more broad-based research and political initiative is needed to enact positive change in Maya women’s lives. Methodologically, our study demonstrates how multi-method approaches are necessary to ensure violent experiences are not underreported. The experiences of these women exemplify the need for rigorous scrutiny of the gender-, race-, and social origin-based dynamics that subject Indigenous women to violence and deprive them of essential post-violence resources. By highlighting their experiences, we hope to encourage interest in this area of research; to identify specific trends and characteristics worthy of further academic attention; to bolster the motivation for eventual policy maneuvers that could improve the life chances of people in such environments; and, ultimately, to promote an active, concrete respect for their dignity through acknowledgement of their unique narratives, perspectives, and needs.
